# Low frequency of *SLC26A4* c.919-2A > G variant among patients with nonsyndromic hearing loss in Yunnan of Southwest China

**DOI:** 10.1186/s12920-024-01829-3

**Published:** 2024-02-20

**Authors:** Yan-Qiong Li, Heng Ma, Qin-Yao Wang, De-Sheng Liu, Wei Wang, Shi-Xin Li, Rong-Xia Zuo, Tao Shen, Bao-Sheng Zhu, Ya-Lian Sa

**Affiliations:** 1 Center for Clinical Medicine Research (Yunnan Provincial Key Laboratory of Clinical Virology, 202205AG070053, L-2019003), The First People’s Hospital of Yunnan Province (The Affiliated Hospital of Kunming University of Science and Technology/School of Medicine), Kunming, 650032 Yunnan China; 2 The Outpatient Department of Yanan Hospital Affiliated to Kunming Medical University, Kunming, 650051 Yunnan China; 3 Central Sterile Supply Department, The First People’s Hospital of Yunnan Province, (The Affiliated Hospital of Kunming University of Science and Technology/School of Medicine), Kunming, 650032 Yunnan China; 4 The Emergency Department of Traditional Chinese Medicine Hospital of Sichuan Province, (The Affiliated Hospital of Chengdu University of Traditional Chinese Medicine), Chengdu, 610075 Sichuan China; 5 Center of Genetic Diagnosis (Yunnan Provincial Key Laboratory for Birth Defects and Genetic Diseases), The First People’s Hospital of Yunnan Province (The Affiliated Hospital of Kunming University of Science and Technology/School of Medicine), Kunming, 650032 Yunnan China

**Keywords:** *SLC26A4*, c.919-2A > G, c.2168 A > G, Gene variant, Nonsyndromic hearing loss, Chinese population

## Abstract

**Background:**

Gene variants are responsible for more than half of hearing loss, particularly in nonsyndromic hearing loss (NSHL). The most common pathogenic variant in *SLC26A4* gene found in East Asian populations is c.919-2A > G followed by c.2168A > G (p.H723R). This study was to evaluate their variant frequencies in patients with NSHL from special education schools in nine different areas of Southwest China’s Yunnan.

**Methods:**

We performed molecular characterization by PCR-products directly Sanger sequencing of the *SLC26A4* c.919-2AG and c.2168 A > G variants in 1167 patients with NSHL including 533 Han Chinese and 634 ethnic minorities.

**Results:**

The *SLC26A4* c.919-2A > G variant was discovered in 8 patients with a homozygous state (0.69%) and twenty-five heterozygous (2.14%) in 1167 patients with NSHL. The total carrier rate of the c.919-2A > G variant was found in Han Chinese patients with 4.50% and ethnic minority patients with 1.42%. A significant difference existed between the two groups (*P* < 0.05). The c.919-2A > G allele variant frequency was ranged from 3.93% in Kunming to zero in Lincang and Nvjiang areas of Yunnan. We further detected the *SLC26A4* c.2168 A > G variant in this cohort with one homozygotes (0.09%) and seven heterozygotes (0.60%), which was detected in Baoshan, Honghe, Licang and Pu`er areas. Between Han Chinese group (0.94%) and ethnic minority group (0.47%), there was no statistical significance (*P* > 0.05). Three Han Chinese patients (0.26%) carried compound heterozygosity for c.919-2A > G and c.2168 A > G.

**Conclusion:**

These data suggest that the variants in both *SLC26A4* c.919-2A > G and c.2168 A > G were relatively less frequencies in this cohort compared to the average levels in most regions of China, as well as significantly lower than that in Han-Chinese patients. These results broadened Chinese population genetic information resources and provided more detailed information for regional genetic counselling for Yunnan.

**Supplementary Information:**

The online version contains supplementary material available at 10.1186/s12920-024-01829-3.

## Background

Hearing loss (HL) is one of the most prevalent disabilities [1]. There is a broad spectrum of genetic and environmental factors involved in hearing loss [2]. Genetic factors are responsible for more than half of cases. The variants of solute carrier family 26 member 4 (*SLC26A4*) gene (MIM #605,646), also named the *PDS* gene, described as the second leading cause after the gap junction protein β 2 (*GJB2*) gene (MIM #121,011), were responsible for autosomal recessive NSHL (DFNB4, MIM #600,791) and syndromic deafness characterized by congenital sensorineural hearing loss, abnormalities of the cochlea, and goiter named as Pendred syndrome (PS, MIM #274,600) [3, 4].

The *SLC26A4* gene, located on chromosome 7q22.q31, contains 21 exons, spans approximately 2343 bp of cDNA and encodes pendrin. Previous research demonstrated that there are hundreds of known variants in the *SLC26A4* gene spreading over all exons and their flanking sequences associated with an increased risk of hearing loss [5–7]. It was reported that the different ethnic groups and geographical origins have their own distinctive variant hotspot of the *SLC26A4* gene with its frequency [8, 9]. In a multiethnic cohort consisting of 117 deaf patients from Turkey (*n* = 45), Mexico (*n* = 11) and Iran (*n* = 61), the most common pathogenic variants of the *SLC26A4* gene were c.1197delT (p. C400Vfs*32) and c.1226G > A (p. R409H) [10]. Adhikary et al. observed that *SLC26A4* gene variants containing c.1087A > G, c.1195 T > C, c.1363A > T, and c.2145G > T in Indian were found in 215 patients with NSHL [11]. In a meta-analysis, the *SLC26A4* c.919-2A > G (rs111033313) in intron 7, c.2168A > G (rs121908362) in exon 19 were the two most common variants in the East Asian population [12]. Particularly, the *SLC26A4* c.919-2A > G variant was the most prevalent in China [12, 13], c.2168A > G was predominant in Japan and Korea [14, 15]. However, published work has not been well documented the variant frequencies of c.919-2A > G and c.2168A > G in patients with NSHL in most areas of Southwest China’s Yunnan.

Yunnan, with a unique natural and geographical environment, is located in China's southwest frontier and lives together 52 ethnic populations [16]. Compared to the eastern regions of China, the information on the frequencies of *SLC26A4* c.919-2A > G and c.2168A > G variants are still not well known in most areas of Yunnan. In this study, we report the variant frequencies of *SLC26A4* c.919-2A > G and c.2168A > G in 1167 patients with NSHL from special education schools in nine different areas of Yunnan, and compared the difference of their frequencies between Han Chinese group and ethnic minority group, among neighboring populations and countries of China. This research enriches the frequency spectrum of the c.919-2A > G and c.2168A > G variants in the Chinese population and contributes to regional genetic counselling and accurate personalized genetic testing.

## Methods

### Subjects

**Fig. 1:**
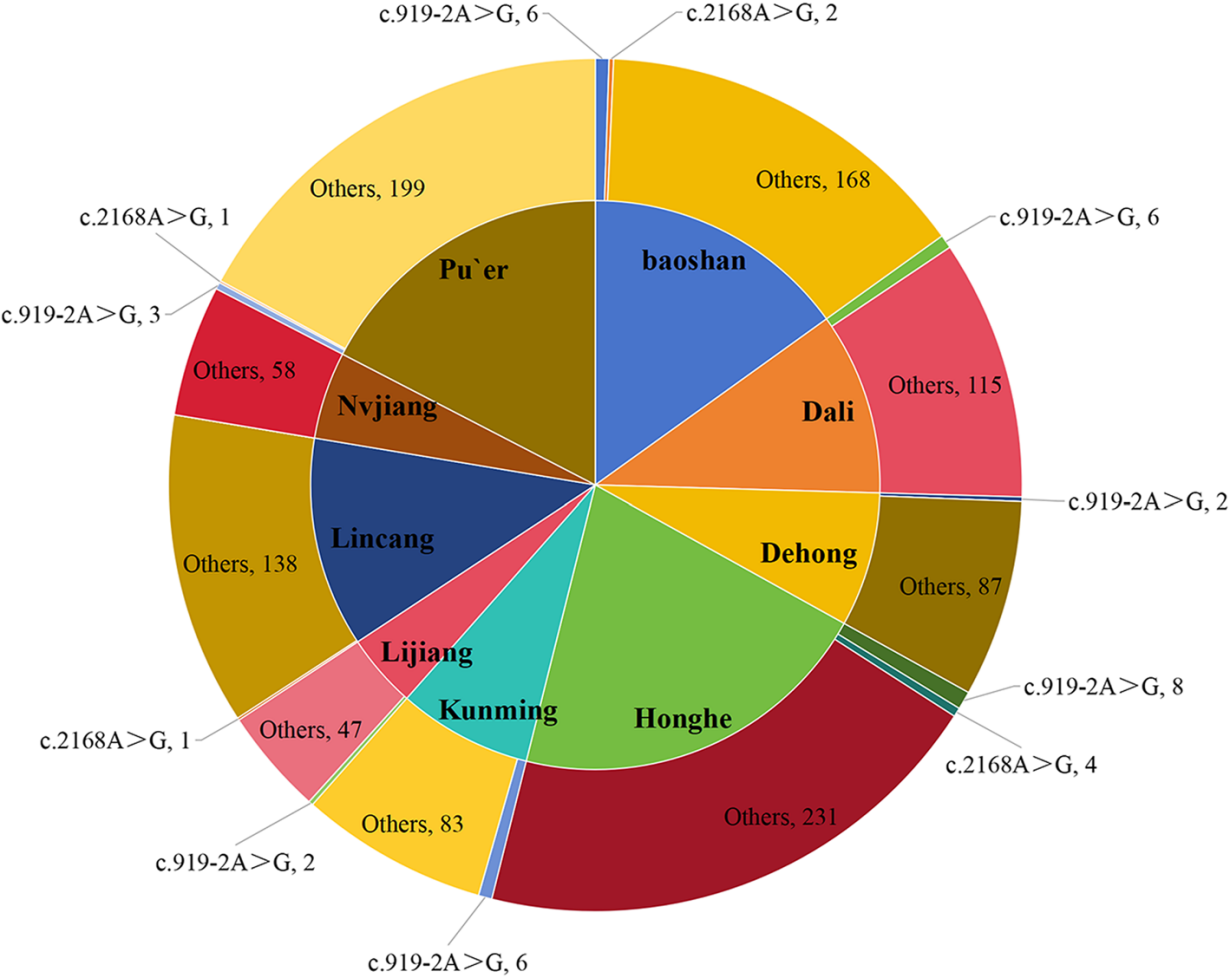
The demographics in nine areas of Yunnan including the counts of participants and *SLC26A4* c.919-2A > G and c.2168A > G variants

A total of 1167 unrelated NSHL patients (604 males, 563 females) from special education schools in nine areas of Yunnan were recruited into our study from January 2011 to October 2013. The subjects with NSHL showed bilateral, mild, and severe to profound, sensorineural hearing loss. This cohort consisted of 634 ethnic minorities (54.33%) and 533 ethnic Han Chinese (45.67%). The median age of the patients with NSHL was 13 years (range, 3–27 years). The basic demographics and the distribution of patients with NSHL in nine geographic areas are provided in Table 1 and Fig. 1 according to the China Sixth Census in 2010. The geographical areas from where individuals were recruited are shown in Fig. 1.

**Table 1:** The demography and the patients with NSHL in nine areas of Yunnan

Name of geographical area	Area (square km)^a^	General population (ten thousand)^a^	ethnic minority population (ten thousand)^a^	Participants with NSHL
China	9,634,057	13,700	904.2	
Yunnan	394,100	4596.6	1533.7	
Kunming	21,473	643.2	88.76	89
Baoshan	19,600	250.6	27.32	176
Dehong	11,173	121.1	55.46	89
Dali	29,459	345.6	182.13	121
Honghe	32,931	450.1	276.81	243
Lijiang	20,600	124.5	70.22	49
Lincang	24,469	243.0	92.10	139
Nvjiang	14,703	53.4	46.83	58
Pu`er	44,221	254.3	155.63	203

^a^These data was from the China Sixth Census in 2010

This study received approval from the Ethics Committee of the First People’s Hospital of Yunnan Province and was performed according to the Declaration of Helsinki of 1975, which was revised in 2008. Written informed consent was obtained from all subjects or their parents/guardians. The patients enrolled in this study were diagnosed with NSHL with a medical certificate and our team-based clinical interview, which excluded syndromic hearing loss and consanguinity, brain injury and meningitis. In this cohort, 94 patients (8.05%) had a family history of deafness.

### PCR amplification and sanger sequencing

All participants donated their 2 ml peripheral venous blood samples and were collected in EDTAK2-containing tubes. Genomic DNA extraction was performed using a Bioteke Blood DNA Extraction Reagent Kit (BioTeke Corporation, Wuxi, China) according to the manufacturer’s instructions. DNA samples were used to detect variants in *SLC26A4* c.919–2 A > G and c.2168A > G by polymerase chain reaction (PCR) amplification followed by Sanger sequencing (ABI 373XL automated DNA sequencer), and analyzed by Genetool software.

### Statistical analysis

Data were analyzed using SPSS statistical software (IBM SPSS Statistics for Windows, Version 19.0.). The significance of differences between groups was assessed by the chi-square test. Fisher’s exact test was used to establish the distributions of genotype and allele. A value of *P* < 0.05 was considered statistically significant. Analysis of data Sequences were compared to the published reference sequence of *SLC26A4* (NG_008489, NM_000441).

## Results

To identify c.919-2A > G and c.2168A > G variants in the *SLC26A4* gene, genomic DNA was extracted, and their regions were amplified by PCR. Amplicons were Sanger sequenced to find c.919-2A > G and c.2168A > G variants.

The distributions of allele and genotypic frequencies of c.919–2 A > G and c.2168A > G in the collected samples were shown in Table 2. Here, we present 1167 patients with NSHL who carried a frequency (2.83%, 33/1167) of *SLC26A4* c.919-2A > G variant, with 8 homozygotes (0.69%) and 25 heterozygotes (2.14%), leading to 1.76% allelic frequency. The graphs of DNA sequencing results for c.919–2 A > G and c.2168A > G were shown in Figure S1.

**Table 2:** The frequencies of *SLC26A4* c.919-2A > G and c.2168 A > G variants between the Han Chinese group and the ethnic minority group in NSHL

Areas	Subjects with NSHL (n)	Subjects with c.919-2A > G, number of cases (%)	Han Chinese with c.919-2A > G,number of cases	Minority patient with c.919-2A > G,number of cases	Homozygous, number of cases (%)	Heterozygous, number of cases (%)	Number of c.919-2A > G variant alleles, frequency(%)
Baoshan	176	6 (3.41)	6	0	1 (0.57)	5 (2.84)	7(1.99)
Dali	121	6 (4.96)	6	0	2 (1.65)	4 (3.31)	8(3.31)
Dehong	89	2 (2.25)	2	0	0(0)	2 (2.25)	2(1.12)
Honghe	243	8 (3.29)	6	2	4 (1.65)	4 (1.65)	12(2.47)
Kunming	89	6 (6.74)	2	4	1 (1.12)	5 (5.62)	7(3.93)
Lijiang	49	2 (4.08)	0	2	0(0)	2 (4.08)	2(2.04)
Lincang	139	0(0)	0	0	0(0)	0(0)	0(0)
Nvjiang	58	0(0)	0	0	0(0)	0(0)	0(0)
Pu`er	203	3 (1.48)	2	1	0(0)	3 (1.48)	3(0.74)
total	1167	33 (2.83)	24	9	8 (0.69)	25 (2.14)	41(1.76)

Ethnically, as shown in Table 2, the *SLC26A4* c.919–2 A > G variant was found in 4.50% (24/533) of Han Chinese patients and 1.42% (9/634) of minority ethnic patients. Of them, the variant frequencies of heterozygotes and the G allele were 3.56% (19/533) and 2.72% (29/1066) in Han Chinese patients, while there were 0.95% (6/634) and 0.95% (12/1268) in minority ethnic patients, respectively. These differences between the two groups were statistically significant (all *P* < 0.05). Homozygotes was detected at a lower rate (0.69%, 8/1167), the frequency of homozygotes (0.94%, 5/533) in the Han Chinese group without statistically significant compared to that (0.47%, 3/634) in the minority ethnic group (*P* = 0.547).

Geographically, a considerable diversity of the *SLC26A4* c.919–2 A > G variant frequency in patients with NSHL was shown in Table 3. In nine areas of Yunnan, the allele frequency of the c.919-2A > G variant was the highest in Kunming with 3.93%, compared with that in other areas. The c.919-2A > G variant was not detected in patients from Lincang and Nvjiang areas.

**Table 3:** The distribution of affected alleles of *SLC26A4* c.919-2A > G was found in patients with NSHL in nine different areas of Yunnan

Gene	Variant	Number of Affected Alleles	Total patients	Han Chinese group	Minority group	χ2	*P*	OR (95%CI)
			**(*n* = 1167)**	**(*n* = 533)**	**(*n* = 634)**			
			(**frequencies**)	(**frequencies**)	(**frequencies**)			
*SLC26A4*	c.919-2A > G	Homozygous	8 (0.69%)	5 (0.94%)	3 (0.47%)	0.363	0.547	1.992 (0.474, 8.373)
		Heterozygous	25 (2.14%)	19 (3.56%)	6 (0.95%)	9.470	0.002	3.869 (1.534, 9.759)
		Total carrier rate	33 (2.83%)	24 (4.50%)	9 (1.42%)	10.018	0.002	3.274 (1.509, 7.107)
		Allele G	41(1.76%)	29(2.72%)	12 (0.95%)	10.562	0.001	2.927 (1.486, 5.765)
	c.2168 A > G	Homozygous	1 (0.09%)	0 (0.00%)	1 (0.16%)	——	1.000	——
		Heterozygous	7 (0.60%)	5 (0.94%)	2 (0.32%)	0.983	0.321	0.334(0.065, 1.729)
		Total carrier rate	8 (0.69%)	5 (0.94%)	3 (0.47%)	0.363	0.547	0.502(0.119, 2.111)
		Allele G	9(0.40%)	5 (0.47%)	4 (0.32%)	0.068	0.794	0.670(0.179, 2.510)

We also detected *SLC26A4* c.2168 A > G variant in this cohort. A summary of its variant frequency was shown in Table 2. Eight patients (0.69%, 8/1167) were found to harbor c.2168 A > G variant involving five Chinese patients (0.43%) and three ethnic minority patients (0.26%), which were one homozygous and seven heterogeneous. Of them, one ethnic minority patient (0.09%) with homozygous was from Pu`er area, and 2 ethnic minority patients with heterogeneous (0.17%) from Honghe area. Five Han Chinese patients with heterogeneous were from Honghe (0.17%, 2/1167), Baoshan (0.17%, 2/1167) and Lincang (0.09%, 1/1167) areas, respectively.

In this cohort, only three Han Chinese patients (0.26%) carried compound heterozygosity for c.919-2A > G and c.2168 A > G, which 2 patients were from Baoshan and one patient in Honghe areas. Above all, *SLC26A4* c.919-2A > G and c.2168 A > G variants were detected at a lower rate among 1167 patients with NSHL in Yunnan compared to the average levels (8.01%, 1.51%) in most of areas of China [17].

The comparisons of the two major pathogenic variant frequencies of *SLC26A4* c.919-2A > G and c.2168A > G among neighboring populations and countries of China, and between the Han Chinese group as well as ethnic minority group in China were shown in Tables 4, and 5, respectively. Generally, the distribution trend of c.919-2A > G variant frequency was higher in Eastern and Central regions than the Western regions, the Han Chinese patients harboring more variant frequency than the Ethnic minority patients with NSHL in China.

**Table 4:** Comparison of the variant frequencies of c.919-2A > G and c.2168 A > G in *SLC26A4* gene in patients from neighboring populations and countries of China

Author Reference	Country /region, area	Samples (n)	c.919-2A > G, No. of allele, frequency (%)	c.2168 A > G, No. of allele, frequency (%)
in this paper	China /Yunnan	1167	41 (1.76%)	9 (0.40%)
Tekin et al., 2003 [18]	Turkey	333	0.00%	2 (0.30%)
Anwar S et al., 2009 [19]	Pakistanis	775 Pakistani families	0.00%	0.00%
Adhikary et al., 2015 [11]	India	215	0.00%	0.00%
Kahrizi et al., 2009 [20]	Iran	80	0.00%	0.00%
Park et al., 2003 [15]	Korea	92	4 (2.17%)	5 (2.72%)
Usami, et al., 1999 [21]	Japan	264	2 (0.38%)	22 (4.17%)
Erdenechuluun et al., 2018 [22]	Mongolia	188	7 (1.86%)	0.00%
Danilchenko et al., 2021 [23]	Russia/Southern Siberia	232	76 (12.14%)	4 (0.64%)
Yuan et al., 2012 [17]	China /27 regions	2352	377 (8.01%)	71 (1.51%)
Wang Y et al., 2021 [24]	China / 3 regions	475	65 (6.84%)	10 (0.98%)
Liu et al., 2016 [25]	China/Inner Mongolia Autonomous Region	738	168 (11.38%)	12 (0.81%)
Yuan et al., 2012 [26]	China/Tibet	114	0.00%	0.00%
Duan et al., 2021 [27]	China/ Qinghai	440	42(4.77%)	17(1.93%)
Pan et al., 2017 [28]	China/Heilongjiang	380	51 (6.71%)	25 (3.29%)
Zhu et al., 2015 [29]	China/Hebei	318	69 (10.85%)	21 (3.30%)
Xie et al., 2021 [30]	China/Hubei	137	24 (8.76%)	4 (1.46%)
Xiang et al., 2019 [31]	China/Zhejiang	506	37 (3.66%)	7 (0.69%)
Lin et al., 2019 [32]	China/Guangdong	634	79 (6.23%)	11 (0.87%)
Huang et al., 2018 [33]	China/Hainan	299	24 (4.01%)	1 (0.17%)
Wu et al., 2019 [34]	China/Taiwan	346	44 (6.36%)	39 (5.64%)
Southwest of China				
Jiang et al., 2015 [35]	China/Chongqing	59	2 (1.69%)	0.00
Dai et al., 2008 [13]	China/Sichuang	109	19 (8.72%)	—
Dai et al., 2008 [13]	China/Guizhou	138	11(3.99%)	—
Samples from the same city of Yunnan				
in this paper	Yunnan/Kunming	89	7 (3.93%)	0.00%
Xin et al., 2013 [36]	Yunnan /Kunming	235	20 (4.26%)	3 (0. 64%)
Dai et al., 2008 [13]	Yunnan /Kunming	159	26 (8.18%)	—
in this paper	Yunnan /Lincang	139	0.00%	1 (0.36%)
Dai et al., 2008 [13]	Yunnan /Lincang	73	2 (1.37%)	—

—Data was not published

**Table 5:** Comparison of the variant frequencies of *SLC26A4* c.919-2A > G and c.2168 A > G among Han Chinese and Ethnic minority patients with NSHL in China

Author Reference			Han Chinese patients (n)		Ethnic minority patients (n)	
	Region/area	Han Chinese patients / Minority patients (n)	c.919-2A > G, No. of allele, frequency (%)	c.2168 A > G, No. of allele, frequency (%)	c.919-2A > G, No. of allele, frequency (%)	c.2168 A > G, No. of allele, frequency (%)
in this paper	Southwestern China’s Yunnan	533 / 634	29 (2.72%)	5 (0.47%)	12 (0.95%)	4 (0.32%)
Duan et al., 2015 [37]	Northwester China’s Ningxia, Qinghai	234 / 250	28(5.98%)	14(2.99%)	24(4.80%)	8 (1.60%)
Du et al., 2014 [38]	Northwester China’s Gansu	1809 / 515	386 (10.67%)	112 (3.10%)	52 (5.05%)	27 (2.62%)
Chen et al., 2011 [39]	Northwester China’s Xinjiang	151 / 199	25 (8.28%)	5 (1.66%)	5 (1.26%)	2 (0.50%)
Dai et al., 2008 [13]	China/27 regions	2783 / 465	533 (9.58%)	——	29 (3.12%)	——

——Data was not published

## Discussion

Hearing loss can be caused by a heterogeneous etiology involving genetic and environmental factors [2, 6]. Previous studies supported the *SLC26A4* gene, especially the c.919-2A > G followed by c.2168A > G variant, play a critical role in molecular etiology of NSHL in the East Asia population [7–9].

In this study, PCR products direct Sanger sequencing was employed to analyze the *SLC26A4* c.919-2A > G and c.2168A > G variants in 1167 patients with NSHL from special education schools in nine different areas of Southwest China’s Yunnan, which included 533 Han Chinese and 634 ethnic minorities. The present study shown that 33 (2.83%) out of 1167 NSHL patients carrying *SLC26A4* c.919-2A > G variant were detected, and eight patients (0.69%) were found to harbor c.2168 A > G variant. Their allele frequencies were significantly lower (1.76%, 0.40%) than the average levels (8.01%, 1.51%) in most regions of China reported by Yuan and his colleagues [17].

The c.919-2A > G variant frequency in this current study was different from those reported by Dai and other researcher [13, 17, 24–38]. Dai and colleague demonstrated that 158 homozygotes (4.83%) and 250 heterozygotes (7.64%) of the *SLC26A4* c.919-2A > G variants were found in 3271 patients with NSHL from 27 regions of China [13]. Its allele frequency varies widely from 19.94% in Henan to 0.40% in Lhasa of Tibet Autonomous, which was 8.18% in Kunming. Xin’s research demonstrated the c.919-2A > G variant frequency was 4.26% in patients from Kunming [35]. Our results shown that the allele frequency of c.919-2A > G variant was found 3.93% in patients from Kunming. The difference of c.919 A > G variant frequency in the patients from the same area was presented. The reason may be partly explained by sample bias.

Previous studies have revealed that homozygous (biallelic variation) or compound heterozygosity for c.919-2A > G and c.2168 A > G in the *SLC26A4* gene was the molecular genetic etiology of sensorineural hearing impairment [9, 11, 12, 15]. In the current study, 8 cases (0.69%) were found to harbor homozygotes of c.919-2A > G, one case (0.09%) with c.2168 A > G homozygotes, and three patients harbored compound heterozygosity for c.919-2A > G and c.2168 A > G. Their variant frequencies were significantly lower compared with the average levels in most regions of China reported by Yuan and Dai et al. [13, 17]. The reasons may be interpreted as follows. First, the c.919-2A > G and c.2168 A > G variants in this cohort may not be the main susceptibility sites to NSHL. Second, there may be small sample bias in this cohort. Third, it can be interpreted in a broader spatial and historical context of population genetics. Yunnan is home not only to Han Chinese but also to 24 officially recognized ethnic minority groups. Populations from different regions of Eurasia as well as Eastern China arrived in Yunnan, and admixed into aborigines with a long history [16]. The present ethnic minority and Han are living together in many places in Yunnan including nine areas in this study, which could have been influenced inter-population marriage. Thus, the homozygote frequency of the *SLC26A4* c.919-2A > G variation can be expected to transiently decline according to the Wahlund principles. Therefore, further studies including a larger number of minority ethnic populations and a broad range of geographic areas are needed in the future.

The c.919 A > G variant was the most prevalent in China, while c.2168A > G variant in Japan and Korea [15, 21]. Zhou et al. demonstrated that 57 patients and 20 patients harbored c.919‐2A > G (4.75%) and c.2168A > G (1.67%) variants were found in 1201 patients with NSHL from Shanxi of China [40]. Chen et al. reported five c.919‐2A > G (0.94%) and three c.2168A > G heterozygous (0.57%) were detected in 530 NSHI patients of south China, including Guangdong, Guangxi, Hainan, Hunan, Fujian and Jiangxi Province [41]. Park HJ et al. demonstrated that one *SLC26A4* c.919-2A > G homozygote (1.09%) and two c.919-2A > G heterozygotes (2.17%) were detected in 92 deaf Korean probands [21]. The frequency of the *SLC26A4* c.919-2A > G allele variant was 0.38% in 264 Japanese individuals [15]. Previous reported that the allele frequency of c.2168A > G variant in patients with NSHL was detected in 2.72%, 4.17%, and 1.51% of Korean, Japanese, and Chinese (mainland Chinese) subjects, respectively [15, 17, 21]. In this study, *SLC26A4* c.919-2A > G and c.2168A > G allele variant in patients from Yunnan were rarely lower frequency (0.40%) than that in patients from neighboring populations and countries of China, except from Tibetan Chinese patients with zero. Thus, these results support the variant frequencies of c.919-2A > G and c.2168A > G in *SLC26A4* gene dependent on the geographical origin [4, 13, 15, 23, 31–33].

Ethnically, the Chinese population consists of 56 ethnic groups. Han Chinese is the largest group. The ethnic minority groups are mainly living in Northwestern and Southwestern of China. Duan et al. reported that the allele frequency of c.919-2A > G was 6.09% (28/460) in Han Chinese patients and 3.33% (14/420) in ethnic minority patients with NSHL from Qinghai in Northwest China [27]. Qing et al. shown that the frequency of the *SLC26A4* variant was 7.04% (82/1164), 7.54% (67/888), 5.43% (15/276) among total subjects, the Chinese Han and ethnic minorities patients in Changsha of Hunan [42], respectively. In this study, ethnic minority patients have a lower carrier frequency (0.95%) than that in Han Chinese (2.72%) with statistical significance. The result was in consistence with the previous research shown in Table 5. Above all, these results support the influence of regional or environmental or ethnic origin on the variant frequency of c.919-2A > G and c.2168 A > G in *SLC26A4* gene [4, 9, 13, 23, 36].

There are some limitations in this study. Even though this project collected 1167 samples from the wide geographic area of Southwest China’s Yunnan, subjects receiving an imaging examination to identify inner ear status or an enlarged vestibular aqueduct (EVA) and temporal bone abnormalities were unclear. Secondly, Sanger sequencing is the gold-standard for all nucleic acid detection. But compared to the next- generation sequencing (NSG) technology, it cannot find more molecular genetic etiology of deafness. Thirdly, there are absence of information on the hearing levels and some other specific clinical features. Thus, possible genotype–phenotype correlations will be to analysis in the future research.

In conclusion, this study demonstrated that the *SLC26A4* c.919-2A > G and c.2168A > G variant frequencies accounted for only a small proportion (1.03%) of patients with NSHL in this cohort. That is, the most common molecular genetic etiology of these patients with NSHL in this cohort is still uncertain. It is probable that hearing loss in these patients is due to variations in other spots in the *SLC26A4* gene or other deafness-related genes. Furthermore, these data would facilitate implementation of the frequency spectrum of the *SLC26A4* c.919-2A > G variant in the NSHL of the Yunnan population. And, it implied that the *SLC26A4* c.919–2 and c.2168A > G are not appropriate for the first step in genetic testing of patients with NSHL in Yunnan.

### Supplementary Information

**Additional file 1: Figure S1. **The chromatograms of the c.919-2A>G and c.2168A>G variant in SLC26A4 gene of Sanger sequencing. A Homozygote. B Heterozygote. C Wild type.

## Data Availability

The data and materials relating to the findings of this study are available from the corresponding author.
