# Supplementary material for: Low frequency of SLC26A4 c.919-2A > G variant among patients with nonsyndromic hearing loss in Yunnan of Southwest China
Source: BMC Med Genomics. 2024 Feb 20;17:55. doi: 10.1186/s12920-024-01829-3 (PMC10877886; doi:10.1186/s12920-024-01829-3)
Supplement: Supplementary file 1 — Additional file 1: Figure S1. The chromatograms of the c.919-2A>G and c.2168A>G variant in SLC26A4 gene of Sanger sequencing. A Homozygote. B Heterozygote. C Wild type. [file 12920_2024_1829_MOESM1_ESM.docx]

**Supplementary Description**

Supplementary Figure 1 (Figure S1) Representation of the chromatograms of the c.919-2A>G and c.2168A>G variants in *SLC26A4* gene.

| **Genotype** | **c.919-2 A>G** | **c.2168A>G** |
| --- | --- | --- |
| A. Homozygote | 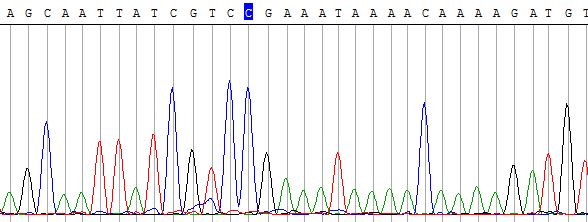 | 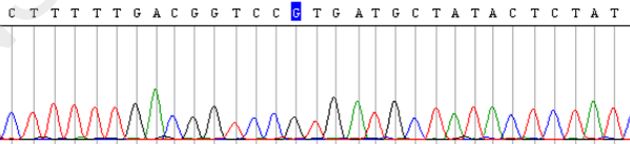 |
| B. Heterozygote | 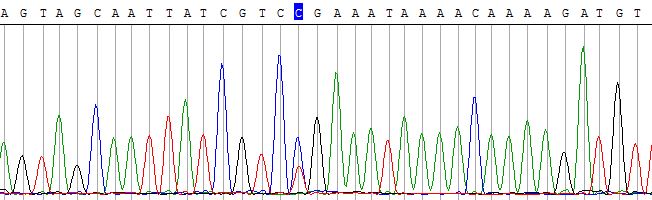 | 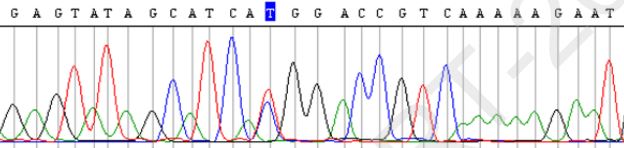 |
| C.  Wild type | 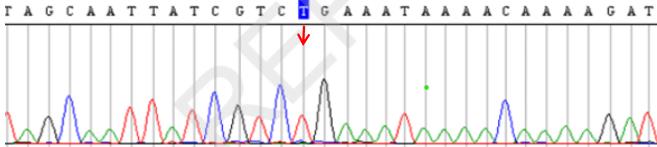 | 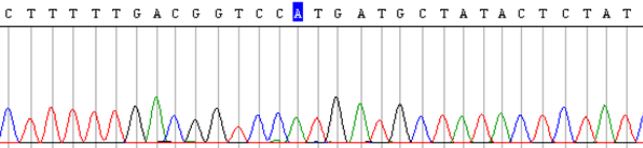 |

Figure S1 is the chromatograms of the c.919-2A>G and c.2168A>G variants in *SLC26A4* gene of Sanger sequencing from patients with NSHL.

A, B and C is homozygote, heterozygote and wild type, respectively.

Nucleic variant sites with red arrow.

*SLC26A4*: solute carrier family 26, member 4.
